# A probabilistic soft-classification framework for estimating forest aboveground carbon stocks with uncertainty in a small-sample mountainous region

**DOI:** 10.3389/fpls.2026.1788418

**Published:** 2026-03-20

**Authors:** Siqi Meng, Chao Zhang, Li Zhou, Zeyu Li

**Affiliations:** 1 College of Forestry, Southwest Forestry University, Kunming, China; 2 Yunnan Key Laboratory of Ecological Environment Evolution and Pollution Control in Mountainous Rural Areas, Southwest Forestry University, Kunming, China; 3 Yunnan Zhan Yi Karst Ecosystem Fixed-Point Observation and Research Station, National Forestry and Grassland Administration, Kunming, China; 4 College of Soil and Water Conservation, Southwest Forestry University, Kunming, China

**Keywords:** AGCS, predictive uncertainty, probability-weighted expectation, soft classification, spatial cross-validation

## Abstract

**Introduction:**

Forest aboveground biomass (AGB) is a critical carbon reservoir, and forest aboveground carbon stock (AGCS) is an important indicator of ecosystem carbon sequestration potential. However, accurate regional-scale AGCS mapping remains challenging in data-scarce regions because sparse field samples and strong spatial heterogeneity can lead to unstable errors that are difficult to quantify in both traditional and machine-learning regression models.

**Methods:**

To address this issue, we developed a Probabilistic Soft-Classification–Expectation Fusion (PSC–EF) framework for small-sample AGCS estimation in Huize County, China. The framework integrates Landsat 5 and ALOS PALSAR imagery with 42 field plots. It first uses a multi-source feature classifier to generate pixel-wise probability maps for three carbon-density classes, then reconstructs a continuous AGCS surface through expectation fusion, and finally quantifies uncertainty using variance propagation and bootstrap resampling.

**Results:**

Under 2×2 spatial block cross-validation, PSC–EF achieved a root mean square error (RMSE) of 182.13 Mg C ha^−1^, a mean absolute error (MAE) of 150.92 Mg C ha^−1^, and a bias of −4.64 Mg C ha^−1^ for continuous AGCS estimation. The probability maps showed a coherent low-to-high carbon gradient, and the total AGCS estimate for Huize County was consistent, with overlapping 95% confidence intervals derived from bootstrap resampling (4.54–5.18 × 10^7^ Mg C) and variance propagation (4.84–4.85 × 10^7^ Mg C).

**Discussion:**

The PSC–EF framework enables continuous AGCS mapping from probabilistic class membership while providing uncertainty information suitable for spatial decision-making. This framework provides spatially explicit carbon stock estimates with quantified uncertainty, supporting local carbon management and offering a transferable approach for AGCS assessment in other data-scarce regions.

## 1 Introduction

In the context of global climate change and the pursuit of “dual carbon” goals, forest ecosystems play a pivotal role. As a major component of the terrestrial carbon pool, they contribute significantly to absorbing and sequestering atmospheric CO_2_, thereby regulating regional and global carbon balances (Pan et al., 2011; Baccini et al., 2012). Aboveground Carbon Stock (AGCS) is a vital indicator of forest structure and productivity, as well as a fundamental variable for assessing forest carbon sink capacity and ecological service value. Consequently, the accurate characterization of spatial AGCS patterns is essential. It enables the quantification of forest contributions to regional carbon budgets, informs ecological restoration and national land-use planning, and provides critical data for evaluating forest carbon sequestration potential and supporting carbon neutrality initiatives (Zhu et al., 2025; Fu et al., 2022).

Recent studies show that satellite remote sensing can capture long-term dynamics of land cover and vegetation, as well as related disturbances, helping disentangle climatic and human influences. For example, MODIS-based analyses have tracked land cover change together with wildfire occurrence and their links to climate variability across Italy since 2001 (Ghaderpour et al., 2025); while cloud-based workflows such as Google Earth Engine have been used to assess multi-year biomass carbon trends at broad scales (Fang et al., 2025). These advances highlight the value of satellite observations for carbon monitoring; however, robust spatial mapping of forest AGCS remains challenging in heterogeneous mountainous regions with limited field data. In recent years, the integration of remote sensing and machine learning has become a mainstream approach for estimating forest AGCS. These approaches can be broadly categorized into three paradigms. The first utilizes multi-source optical, synthetic aperture radar (SAR), and topographic data to establish pixel-scale regression or ensemble learning models for direct prediction of continuous biomass or carbon density (Khan et al., 2024; Liang et al., 2025; Tian et al., 2024). The second focuses on remote sensing-based forest stand stratification or sample expansion, estimating regional totals by applying the mean carbon density of each stratum to its corresponding area (Fu et al., 2025). The third paradigm integrates vegetation indices with microwave vegetation optical depth (VOD) at broader scales, employing algorithms such as random forests to reconstruct spatiotemporal dynamics of aboveground carbon (Chang et al., 2023). Collectively, these studies demonstrate the significant potential of multi-source remote sensing and machine learning for forest carbon stock estimation. However, their performance is generally contingent upon the availability of sufficient ground plot data and relatively homogenous environmental conditions (Ma et al., 2024). Consequently, in regions constrained by limited sample sizes and highly heterogeneous topography and forest structure, ensuring the robustness, interpretability, and uncertainty quantification of AGCS estimates remains a prominent research challenge.

Hard classification assigns a single, discrete category label to each pixel (Feilhauer et al., 2021). In forest AGCS inversion, this leads to a common “classify-then-estimate” paradigm: following classification, a fixed average carbon density value is assigned to all pixels within a category, or separate empirical models are developed per category to complete the estimation (Cheng et al., 2024). While this approach offers advantages in simplicity and interpretability, its decision boundaries are often contingent on subjective (user-defined) thresholds and limited sample data. A key limitation arises in ecotones such as forest edges, topographic transition zones, and fragmented patches. Here, mixed pixels—containing multiple land cover types—are forcibly assigned to a single class. This process compresses the intrinsic spectral and biophysical variability within a pixel into a single, discrete label. Consequently, this can generate unrealistic spatial discontinuities in carbon stock maps and introduce systematic estimation bias (Enterkine et al., 2024; Luo et al., 2024; Martinez-Sanchez et al., 2024). These limitations are particularly pronounced in mountainous regions, which are typically characterized by sparse ground samples and high spatial heterogeneity. Under such conditions, the extrapolation stability and predictive accuracy of hard classification-based methods are severely constrained.

In contrast to hard classification, soft (probabilistic) classification forgoes assigning a single, definitive label to each pixel. Instead, it estimates posterior probabilities (or membership grades) across all predefined classes, quantifying the likelihood of a pixel belonging to various cover types or AGCS levels (Vishnoi and Pareek, 2025). For example, a pixel might be characterized by posterior probabilities of 0.2, 0.5, and 0.3 for Low, Mid, and High biomass levels, respectively. This directly reflects its mixed compositional nature and captures sub-pixel scale transitional characteristics. Previous research underscores the utility of this approach. For instance, Xu et al (Xu et al., 2005). implemented soft classification via decision tree regression, demonstrating that probabilistic outputs can significantly mitigate estimation fluctuations caused by limited training samples. Separately, Pontius and Cheuk (Pontius and Cheuk, 2006) proposed the generalized contingency matrix, providing a unified framework for assessing the accuracy of soft-classification probability maps against reference data. Consequently, in forest and ecological remote sensing, class probability maps and their derived uncertainty metrics have become standard outputs for tasks ranging from forest type classification and biomass estimation to the cartographic representation of spatial uncertainty (Foody and Mathur, 2006). Building on this foundation, a natural bridge between categorical mapping and continuous-variable estimation can be constructed. This is achieved by fusing the probabilities of carbon-density levels with their level-specific representative value to retrieve a continuous AGCS estimate via a probability-weighted expectation.

Despite recent advances in remote sensing and machine learning, robust mapping of forest aboveground carbon stock (AGCS) remains challenging in data-scarce mountainous regions, where field plots are sparse, landscape heterogeneity is high, and uncertainty is often not explicitly characterized at pixel and regional scales. To address these challenges, this study focuses on Huize County in Yunnan Province, China, and integrates Landsat 5 optical imagery, ALOS PALSAR radar data, and 42 forest continuous inventory (FCI) plots to develop a continuous AGCS estimation framework suitable for small-sample and topographically complex settings. The proposed framework couples probabilistic soft classification and expectation fusion with uncertainty quantification, and the main contributions are:

- A lightweight probabilistic soft-classification model is constructed for three carbon-density tiers (Low, Mid, High) and evaluated using spatial block cross-validation to derive pixel-wise posterior probability maps.
- Posterior probabilities are fused with class-specific representative carbon densities through a probability-weighted expectation (PWE) scheme to reconstruct a continuous AGCS surface.
- Uncertainty is quantified at both pixel and regional scales by combining tiered variance propagation with bootstrap resampling.

The remainder of this paper is organized as follows: Section 2 presents the materials and methods, Section 3 reports the results, Section 4 discusses the findings and associated uncertainties, and Section 5 concludes the paper.

## 2 Materials and methods

### 2.1 Study area

**Figure 1:**
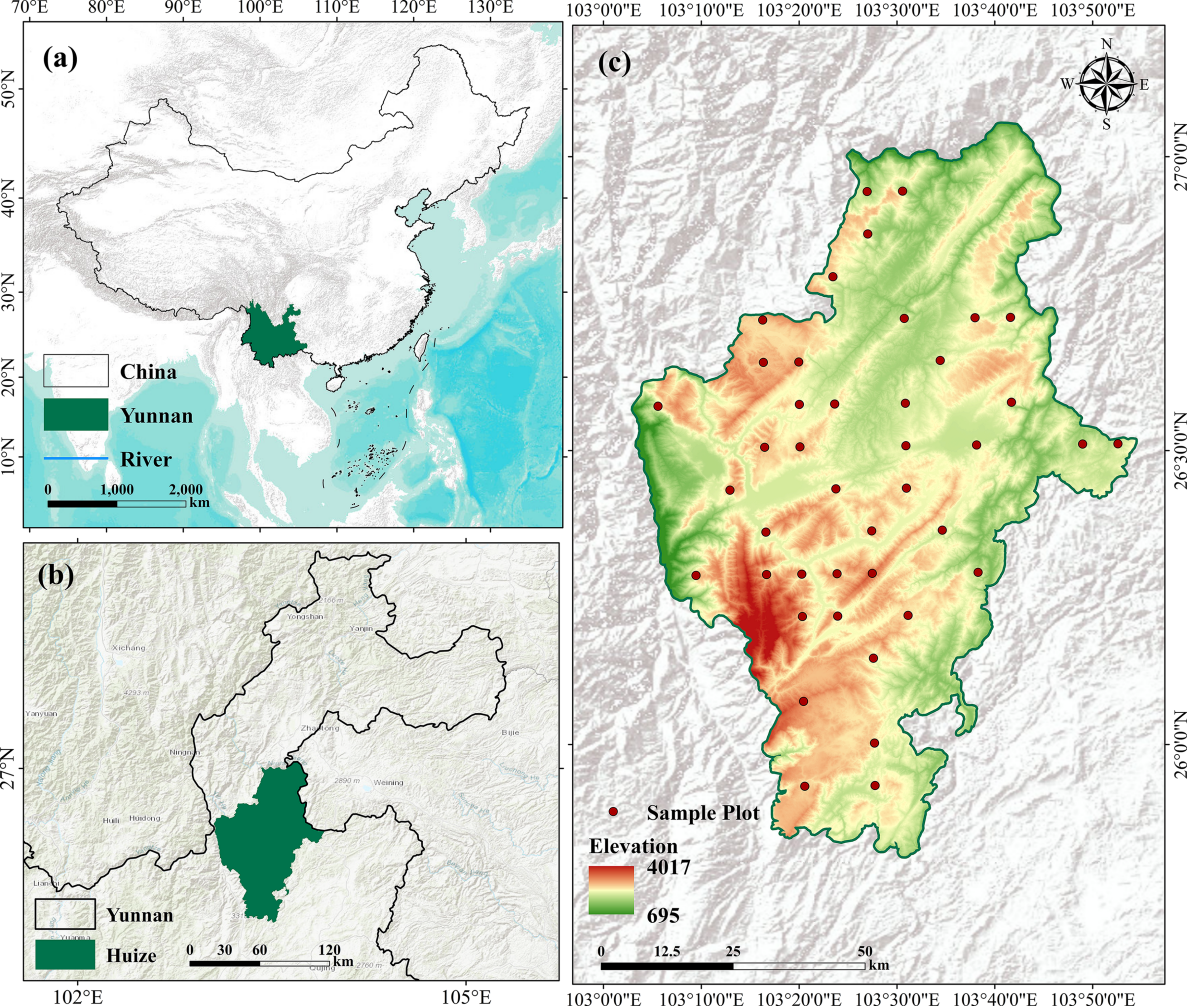
Schematic map of the study area. **(a)** shows a map of China (Map Approval No.: GS(2024)0650), **(b)** indicates the geographical location of Huize County within Yunnan Province, and Figure **(c)** presents the digital elevation model (DEM) of Huize County with the locations of the field sampling plots.

The study area, Huize County, is situated within Qujing City in Yunnan Province, southwestern China (25°27′–27°04′ N, 102°43′–104°14′ E). It lies in the Wumeng Mountains on the northeastern Yunnan Plateau. Encompassing approximately 5800 km^2^, the county’s terrain is predominantly mountainous (>95%), featuring pronounced topographic relief and deeply incised valleys. Elevation ranges from 700 to 4017 meters above sea level (m a.s.l.), creating a distinct stepped topography that descends from west to east (**Figure 1**). The region experiences a subtropical montane monsoon climate, shaped by low-latitude plateau atmospheric circulation and pronounced elevational gradients. The mean annual temperature is approximately 13 °C, and the mean annual precipitation is around 800 mm, with the majority concentrated during the monsoon season from June to September. The forest cover is dominated by mixed conifer–broadleaf and pure conifer stands. Prominent tree species include *Pinus armandii*, *Pinus yunnanensis*, various oaks (*Quercus* spp.), and *Cupressus* spp. The interplay between complex topography and heterogeneous forest composition creates strong spatial heterogeneity in AGB This makes Huize County a representative and challenging testbed for developing and evaluating continuous AGCS estimation methods under data-scarce conditions.

### 2.2 Data collection and processing

Multiple datasets, including field inventory plots, optical imagery, radar backscatter, and topographic data, were used in this study (**Table 1**).

**Table 1:** Summary of datasets used in this study.

Dataset	Spatial resolution	Temporal resolution	Period used	Data source
Forest plots (FCI)	Plot scale (0.08 ha)	One campaign	2007	Seventh Forest Resources Continuous Inventory (FCI) of Yunnan Province (2007) (no public DOI/link)
Landsat 5 TM	30 m	16 days	2007-01-01–2007-12-31	https://developers.google.com/earth-engine/datasets/catalog/LANDSAT_LT05_C02_T1_L2
ALOS PALSAR	resampled to 30 m	Annual mosaic	2007	https://developers.google.com/earth-engine/datasets/catalog/JAXA_ALOS_PALSAR_YEARLY_SAR
SRTM DEM	resampled to 30 m	Static	—	https://developers.google.com/earth-engine/datasets/catalog/USGS_SRTMGL1_003

GEE dataset IDs and catalog links are provided for reproducible access; the field inventory dataset has no public DOI/link.

#### 2.2.1 Plot data

The forest plot data used in this study were derived from the Seventh Forest Resources Continuous Inventory (FCI) of Yunnan Province (2007). The inventory employed a systematic sampling grid of 6 km × 8 km, with each sample plot covering an area of 0.08 ha. A total of 42 plots were selected within Huize County, focusing on forest stands dominated by locally prevalent tree species or species groups. These plots were used for subsequent biomass and carbon stock calculations (see **Table 2**). AGB was calculated using a species-specific volume-biomass model. Subsequently, AGCS was derived by applying a biomass-carbon conversion factor to the estimated AGB. The specific models are presented below:

(1) B=aV+b

(2) C=B×Cc

In Equation 1, B is forest biomass, Mg ha^-^¹; V is forest volume, m^3^/ha; a, b is the model conversion parameter between volume and biomass, using the conversion parameters proposed by Fang et al (Fang et al., 1998). for volume-to-biomass conversion. In Equation 2, C is Carbon storage, Mg C·ha^-1^ (megagrams of carbon per hectare); Cc is Carbon content coefficient.

**Table 2:** Model conversion parameters for key advantageous tree species in Huize County.

No.	Dominant tree species	Parameter a	Parameter b	Carbon fraction ( Cc)
1	*Cupressus funebris* Endl	0.6129	46.1415	0.5034
2	*Pinus armandii* Franch.	0.5856	18.7435	0.5225
3	*Quercus* spp.	1.1453	8.5457	0.5004
4	Soft broadleaved species	0.4754	30.6034	0.4956
5	*Populus* spp.	0.0849	0.8710	0.4705
6	*Keteleeria fortunei* (A. Murray bis) Carrière	0.4158	41.3318	0.4997
7	*Pinus yunnanensis* Franch.	0.5101	1.0451	0.5113
8	*Mixed coniferous and broad-leaved forest*	0.7143	16.9654	0.4978
9	*Mixed coniferous forest*	0.5894	24.5151	0.5101

#### 2.2.2 Landsat5 TM data

The primary optical data source was imagery acquired by the Landsat 5 Thematic Mapper (TM) sensor from the United States Geological Survey (USGS) Landsat Collection 2 Level-2 Surface Reflectance product. All scenes acquired from January to December 2007 covering the study area were processed on the Google Earth Engine (GEE) platform. Landsat 5 TM provides 30 m observations in the visible, near-infrared, and shortwave infrared regions. For this analysis, reflective bands 1–5 and 7 were selected as primary spectral inputs, while the thermal band (band 6) was excluded. Surface reflectance was obtained directly from the Level-2 product, and cloud screening was implemented using the QA_PIXEL quality assessment band, whose cloud information is produced within the USGS QA framework (Zhu and Woodcock, 2012). Scenes were filtered by metadata (CLOUD_COVER ≤ 20%), and pixels flagged in QA_PIXEL as Cloud (bit 3) or Cloud Shadow (bit 4) were masked to remove cloud-contaminated observations. After metadata filtering and QA-based masking, a per-pixel median composite was generated from the remaining clear observations to produce a single cloud-screened surface reflectance image at 30 m resolution for 2007. Reflectance scale factors were applied to the surface reflectance bands (multiplying by 0.0000275 and adding −0.2) before compositing, following the Landsat Collection 2 Level-2 specification. The 2007 composite ensures temporal consistency with the 2007 field survey and provides stable optical inputs for vegetation index calculation and feature extraction (Zhu et al., 2015; Gorelick et al., 2017).

#### 2.2.3 ALOS PALSAR data

Radar data were accessed on the Google Earth Engine (GEE) platform from the Japan Aerospace Exploration Agency (JAXA) Advanced Land Observing Satellite (ALOS) Phased Array type L-band Synthetic Aperture Radar (PALSAR) yearly mosaic image collection, which provides annual global mosaics of PALSAR observations. Dual-polarization observations (HH and HV) for 2007 were selected from the annual mosaic, and the radar layers were resampled to a 30 m grid to match the spatial resolution of the optical and terrain datasets. The yearly mosaic stores polarization layers as 16-bit digital numbers (DN), which can be converted to gamma-naught backscatter using the dataset-provided formulation. Following the dataset specification, DN values were converted to linear $\sigma^{0}$as $\sigma^{0}=$DN^2^ /10^8.3^, and non-positive DN values were masked prior to conversion. Speckle noise was reduced in the linear domain using a Refined Lee (local-statistics) filter, and filtered HH, HV, and the HH/HV ratio were then derived (Lee, 1981). In the Refined Lee filtering, the equivalent number of looks was set to L = 5, and the HH/HV ratio was used as a structural indicator related to canopy properties and biomass gradients (Shimada et al., 2010, Shimada et al., 2014).

#### 2.2.4 DEM data

Terrain information was derived from the Shuttle Radar Topography Mission (SRTM) Digital Elevation Model (DEM) (1 arc-second, ~30 m), resampled to a 30 m grid. The DEM was accessed through the GEE platform and clipped to the boundary of the study area. Using spatial analysis tools in ArcGIS, three primary terrain factors—elevation, slope, and aspect—were calculated from the DEM. These factors are key for characterizing local hydrothermal conditions and understanding forest spatial patterns influenced by topography. To ensure spatial consistency across all datasets, the optical, radar, and DEM layers were reprojected to a common coordinate system, resampled to a uniform 30-meter grid, and co-registered. This rigorous geometric correction established a consistent spatial framework essential for accurate feature extraction from sample plots and for all subsequent integrative modeling (Horn, 1981; Farhadpour et al., 2024).

### 2.3 Research methods

**Figure 2:**
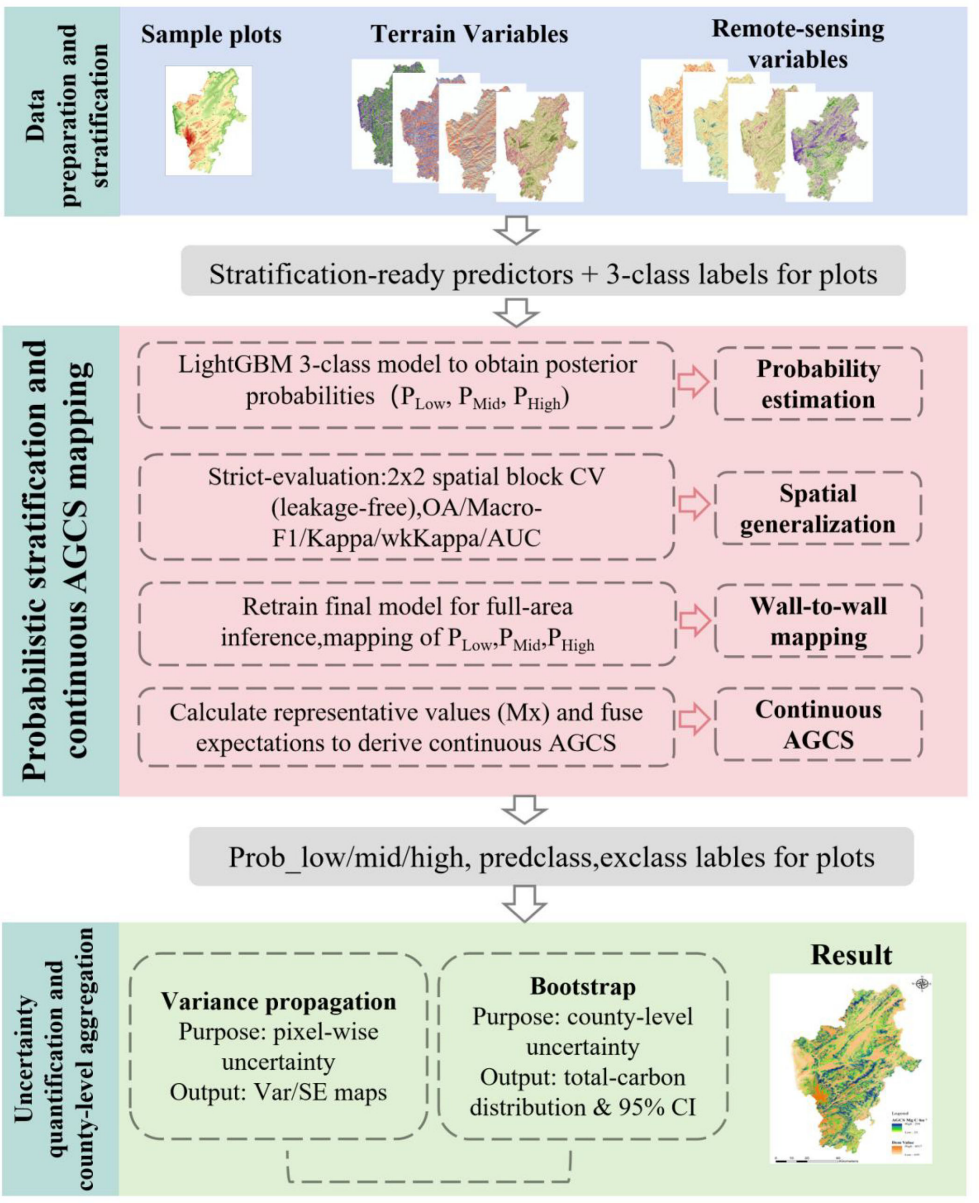
Technical route.

To enable continuous estimation of forest AGCS in Huize County under conditions of limited sample size and pronounced topographic heterogeneity, this study developed a methodological framework termed Probabilistic Soft-Classification-Expectation Fusion (PSC–EF), based on multi-source remote sensing features. The overall technical workflow is shown in **Figure 2**. It consists of four main stages: (1) data preprocessing and feature engineering; (2) lightweight three-class modeling and spatial block cross-validation; (3) continuous AGCS retrieval via probability-weighted expectation; and (4) uncertainty assessment and regional total estimation integrating variance propagation and bootstrap resampling.

#### 2.3.1 Data preprocessing and feature construction

A multi-source feature set was constructed from Landsat 5 TM, ALOS PALSAR, and SRTM DEM data to support the inversion of forest AGCS in Huize County. To ensure spatial consistency, all optical, radar, and topographic rasters were reprojected to a common coordinate system, resampled to a 30-meter resolution, and co-registered. Feature values were then extracted from pixels corresponding to the 42 plot locations, along with their local neighborhood statistics, to form the integrated feature set. Optical features comprised surface reflectance from TM bands 1–5 and 7, as well as a suite of vegetation indices: the normalized difference vegetation index (NDVI), enhanced vegetation index (EVI), soil-adjusted vegetation index (SAVI), green normalized difference vegetation index (GNDVI), and modified soil-adjusted vegetation index (MSAVI). To capture local spatial heterogeneity, the mean and standard deviation of key bands and indices were calculated within a sliding window. Radar features included the HH and HV backscatter coefficients, their ratio (HH/HV), and Gray-Level Co-occurrence Matrix (GLCM) texture metrics (e.g., entropy, homogeneity, contrast) derived from the HH polarization channel within the same window. Topographic features encompassed elevation, slope, aspect, and their trigonometric transformations (northness, eastness), which were further synthesized into a composite TopoGroup variable to represent integrated site gradients. Prior to model training, all numerical features underwent preprocessing, including missing value imputation and z-score standardization, to ensure scale comparability across different feature dimensions. The detailed formulations for each vegetation index and texture metric are provided in **Table 3**.

**Table 3:** Calculation methods for vegetation indices and texture parameters.

Types	Name	Formula
Vegetation index	NDVI (Rouse et al., 1974)	$\frac{B_{4}\;-\;B_{3}}{B_{4}\;+\;B_{3}}$
	DVI (Richardson and Weigand, 1977)	$B_{4}\;-\;B_{3}$
	EVI (Miura et al., 2002)	$2.5\times (\frac{B_{4}\;-\;B_{3}}{B_{4}\;+\;(6\times B_{3\;}-\;7\times B_{1})\;+\;1})$
	GNDVI (Daughtry et al., 1992)	$\frac{B_{4}\;-\;B_{2}}{B_{4}\;+\;B_{2}}$
	MSAVI (Qi et al., 1994)	$\frac{2\times B_{4\;}+\;1\;-\;\sqrt{{\left(2\times B_{4\;}+\;1\right)}^{2\;}-\;8\times (B_{4\;}-\;B_{3})}}{2}$
	RVI (Pearson and Miller, 1972)	$\frac{B_{4}}{B_{3}}$
	SAVI (Jackson et al., 1980)	$\frac{\left(B_{4}\;-\;B_{3})(1\;+\;L\right)}{(B_{4\;}+\;B_{3})\;+\;L}$
Textural features	Mean	$\sum_{i,j=0}^{n\;-\;1}iF_{i,j}$
	Variance	$\sum_{i,j=0}^{n\;-\;1}iF_{i,j}{\left(i,j\;-\mu_{i,j}\right)}^{2}$
	Homogeneity	$\sum_{i,j=0}^{n\;-\;1}i\frac{F_{i,j}}{1+{\left(i-j\right)}^{2}}$
	Entropy	$\sum_{i,j=0}^{n\;-\;1}iF_{i,j}\left|-\ln F_{i,j}\right|$
	Correlation	$\sum_{i,j=0}^{n-1}F_{i,j}\left[\frac{\left(i-\mu_{i}\right)\left(j-\mu_{j}\right)}{\sqrt{VA_{i}VA_{j}}}\right]$
	Dissimilarity	$\sum_{i,j=0}^{n\;-\;1}iF_{i,j}\left|i-j\right|$
	Contrast	$\sum_{i,j=0}^{n\;-\;1}iF_{i,j}{\left(i-j\right)}^{2}$
	Angular Second Moment	$\sum_{i,j=0}^{n\;-\;1}iF_{i,j}^{2}$

1) B1, B2, B3, and B4 represent the blue, green, red, and near-infrared bands of the Landsat 5 satellite, respectively; 2) L denotes the soil adjustment factor, with L = 0.5; 3) i and j are the row and column indices of the Gray-Level Co-occurrence Matrix (GLCM), respectively; $F_{i,j}$ represents the value of the element in the normalized symmetric GLCM at row i and column j, and $\mu_{i,j}$ denotes the mean of the GLCM (Wei et al., 2025).

#### 2.3.2 Probabilistic soft classification model

To address the challenges of extremely limited samples and high topographic heterogeneity, this study adopts a Light Gradient Boosting Machine (LightGBM) classifier to perform probabilistic soft-classification of forest aboveground carbon density into three distinct tiers. First, the AGCS values from the 42 sample plots were divided into Low, Mid, and High tiers using quantile thresholds to ensure a balanced distribution of samples across classes. Subsequently, a LightGBM-based multiclass classifier was trained using the multi-source remote sensing and topographic features as predictors and the three carbon-density tiers as the target variable.

LightGBM retains strong nonlinear fitting capability while requiring relatively few hyperparameters and offering high computational efficiency, rendering it well-suited for capturing multi-dimensional feature relationships under small-sample conditions (Ke et al., 2017). This study employs a Softmax output layer, representing the posterior probabilities of each sample plot belonging to the low, medium, and high categories as ( p_Low_, p_Mid_, p_High_), Let the model’s raw scores for the three categories be $f_{k}\left(x\right)$( k∈ {Low, Mid, High}). The posterior probabilities are calculated using the Softmax function in Equation 3, where p_k(x) denotes the posterior probability that sample x belongs to class k, f_k(x) is the model's raw score for class k, and j indexes all classes.

(3) $$p_{k}\left(x\right)=\frac{\text{exp}\left(f_{k}\left(x\right)\right)}{\sum_{j}\text{exp}\left(f_{j}\left(x\right)\right)}$$

This probability vector not only captures the discrete class assignment but also supplies the essential probabilistic basis for the subsequent continuous inversion via probability weighting. The class−specific representative values μ_Low_, μ_Mid_, μ_High_ are defined as the median AGCS of the plots within each class, a choice that reduces the influence of extreme values on the representation of central tendency (Feilhauer et al., 2021; Vishnoi and Pareek, 2025).

While the three-class classifier in this study estimates posterior probabilities for each class label, their architectures lack explicit ordinal constraints. Instead, ordinality is incorporated during the continuous inversion stage through an ordered representative value–probability expectation fusion scheme: the representative values μ_Low_, μ_Mid_, μ_High_ derived from the training plots, satisfy  μ_Low_ <μ_Mid_ <μ_High_, and continuous AGCS is reconstructed accordingly (see Section 2.3.4). This design ensures that as the pixel-level probability mass shifts from Low to High, the resulting continuous estimates follow a numerically consistent gradient in carbon density. The consistency between predicted class order and actual carbon gradient is further evaluated in the Results section by integrating (i) the monotonic increase in AGCS distributions across predicted classes, and (ii) the cross-class misclassification patterns revealed in the confusion matrices.

In the inversion step, a monotonicity constraint ( μ_Low_, μ_Mid_, μ_High_) is explicitly imposed on the median carbon densities used as representative values for the three tiers. This ensures that the resulting class-to-continuous mapping is both numerically and ecologically consistent with the underlying carbon−density gradient. By enforcing this order, the method prevents physically implausible outcomes, such as assigning plots with higher carbon stocks to classes with lower representative values (Israeli et al., 2019; Wang et al., 2022). The outputs of this probabilistic soft-classification model form the basis for spatial mapping and uncertainty assessment.

#### 2.3.3 Spatial cross-validation and performance metrics

To objectively evaluate model performance under spatial extrapolation conditions, a 2×2 spatial block cross-validation (CV) scheme was implemented based on plot coordinates. The study area was divided into four non-overlapping spatial blocks by bisecting the longitude and latitude ranges of the plot distribution. In each CV fold, one block was held out as the test set, while the other three blocks were used for model training. This process was repeated four times so that each block served as the test set once. To prevent information leakage, all preprocessing steps-including missing−value imputation, feature standardization, and one−hot encoding of the TopoGroup variable-were performed independently within each training fold. The resulting transformation parameters were then applied solely to the corresponding test fold (Ploton et al., 2020; Karasiak et al., 2022; Stock, 2025). This design effectively reduces spatial autocorrelation between training and test data, avoids the spatial leakage that can occur under random splitting, and thus provides a more realistic estimate of model extrapolation capability for actual mapping. Model performance is comprehensively characterized using Overall Accuracy (OA), Macro-F1, Cohen’s kappa, weighted kappa, and the AUC computed for both the Low and High one-vs-rest tasks. OA reflects overall correctness but can be biased toward dominant categories under class imbalance. Macro-F1 is computed by calculating the F1 score for each class separately and then taking their arithmetic mean:

(4) $$Macro-F1=\frac{1}{K}\sum_{k=1}^{K}\text{F}1_{k}$$

In Equation 4, where K=3 denotes the number of classes, and $\text{F}1_{k}$ is the F1-score for the k-th class. This metric provides a more balanced assessment of the model’s ability to identify each individual carbon-density level.

Cohen’s Kappa is used to measure the improvement in classification results relative to random agreement:

(5) $$k=\frac{p_{o}-p_{e}}{1-p_{e}}$$

In Equation 5, where ${\text{p}}_{\text{o}}$ denotes the observed agreement (overall accuracy), and ${\text{p}}_{\text{e}}$ represents the expected agreement by chance based on marginal distributions. The quadratic-weighted Kappa coefficient extends this by incorporating a penalty matrix, which assigns higher penalties to serious misclassifications, thereby providing a more sensitive measure of classification performance for ordinal variables (Feizizadeh et al., 2022; Yilmaz and Demirhan, 2023; Farhadpour et al., 2024).

Given the study’s emphasis on identifying extreme low-carbon and high-carbon regions, separate one-vs-rest binary classification tasks were constructed for the Low and High classes (Low vs. rest; High vs. rest). The area under the receiver operating characteristic curve (AUC) was then calculated to quantify the discriminative ability of the model across different classification thresholds (Richardson and Weigand, 1977; Airola et al., 2019). In the Results section, mid-class separability and the ordinal (Low–Mid–High) confusion structure are primarily assessed using Macro-F1, (quadratic-weighted) Kappa, and the AUC for the extreme classes, complemented by a detailed analysis of the confusion matrix. To examine robustness to feature redundancy, two leakage-free within-fold feature screening settings (Corr-pruned 0.98 and PermTop-20) were additionally evaluated alongside the full feature set; the full feature set was retained for the main analyses.

At the continuous-estimation stage, we construct a hard-assignment baseline that is directly comparable to the main method for out-of-fold evaluation. All strategies use the same spatial block partitioning and the same fold-wise preprocessing, and the two classification-based strategies share the same LightGBM three-class classifier and feature set. The continuous-estimation stage is implemented in three parallel ways using the same predictor set, the same 2×2 spatial block partitioning, and the same fold-wise preprocessing: the main method performs probability–expectation fusion using the posterior probability vector ( p_Low_, p_Mid_, p_High_), the hard-assignment baseline assigns the most probable class and its corresponding representative value, and a direct regression baseline (LightGBM regressor) predicts continuous AGCS directly from the predictors. To prevent information leakage and ensure rigorous spatial extrapolation evaluation, ${\text{μ}}_{\text{k}}$ is estimated solely from the training samples within each fold and then applied when generating continuous predictions for the corresponding test fold. The three continuous strategies are compared on out-of-fold samples using RMSE, MAE, and bias, where RMSE and MAE denote the root mean square error and mean absolute error, respectively, Bias represents the mean error (prediction minus observation), serving as a measure of overall systematic overestimation or underestimation.

(6) $$\;\text{RMSE}=\sqrt{\frac{1}{n}\sum_{i=1}^{n}{\left({\hat{C}}_{i}-C_{i}\right)}^{2}}$$

(7) $$\;\text{MAE}=\frac{1}{n}\sum_{i=1}^{n}\left|{\hat{C}}_{i}-C_{i}\right|$$

(8) $$\text{Bias }=\frac{1}{n}\sum_{i=1}^{n}\left({\hat{C}}_{i}-C_{i}\right)$$

In Equations 6–8, n is the number of validation samples, $C_{i}$ and ${\hat{C}}_{i}$ denote the observed and predicted AGCS for plot i, respectively (in the same units as carbon density). RMSE and MAE quantify the overall magnitude of prediction errors, whereas Bias, defined as the mean error ( $\hat{C}-C$), indicates systematic overestimation or underestimation.

#### 2.3.4 Probability-weighted continuous AGCS retrieval

After obtaining the posterior probability fields for the Low, Mid, and High carbon-density classes, we treat the three-class model as an estimator of the conditional probabilities of the latent class variable. On this basis, we use probability-weighted expectation to reconstruct a continuous AGCS field. Let the posterior probabilities that a pixel belongs to the Low, Mid, and High classes given the multi-source feature vector X(x) be denoted as ${\text{p}}_{\text{Low}}\text{(x)}$, ${\text{p}}_{\text{Mid}}\text{(x)}$, ${\text{p}}_{\text{High}}\text{(x)}$, respectively. The corresponding class medians of carbon density, computed from the training plots, are μ_Low_, μ_Mid_, μ_High_. As shown in Equation 9, the pixel-level AGCS estimate is obtained as the probability-weighted expectation of the class-specific median carbon densities (Orhobor et al., 2023; Camara et al., 2024):

(9) $$C\left(x\right)=p_{Low}\left(x\right)\mu_{Low}\;+\;p_{Mid}\left(x\right)\mu_{Mid}\;+\;p_{High}\left(x\right)\mu_{High}$$

Since  μ_Low_ <μ_Mid_ <μ_High_, the expectation-fusion estimator ensures that, as pixel-level probabilities shift from lower to higher grades, $\hat{C}\left(x\right)$ increases monotonically. This enables a smooth mapping from discrete grades to a continuous carbon-density gradient.

Statistically, this estimator treats the class-specific representative values as typical carbon-density intensities under different states and the posterior probabilities as their corresponding weights, thereby approximating a continuous AGCS surface through soft classification without relying on direct continuous regression. Ecologically, this formulation allows a pixel to share weights probabilistically among the Low, Mid, and High classes, making it particularly suitable for capturing carbon-storage gradients in mixed-species stands and transition zones (Feilhauer et al., 2021). Within this probability-weighted framework, the three-class probabilities and the resulting expected AGCS further serve as inputs for subsequent uncertainty assessment and regional total estimation.

#### 2.3.5 Uncertainty assessment

To quantify uncertainty, this study integrates two complementary approaches at distinct spatial scales: variance propagation for pixel-level error structure and bootstrap resampling for regional-total stability.

Variance propagation analytically derives the pixel-level AGCS variance based on the class statistics and the posterior probability maps. Each class (Low, Mid, High) is treated as a conditional distribution characterized by its representative value( $\mu_{k\;},\sigma_{k}$)and within-class standard deviation, estimated from the training plots. The posterior probability $\;\;p_{k}(x)$of a pixel belonging to each class serves as the mixing weight (Araza et al., 2022; David et al., 2024). Within this framework, total pixel−level variance is decomposed into two components: within-class variance and between−class variance, the latter arising from classification uncertainty. Pixel−level variances are then aggregated to the regional scale, assuming negligible spatial covariance among pixels, to yield the variance and standard error of the total AGCS estimate (Qiu et al., 2024). Because spatial covariance is difficult to estimate reliably at the county scale and may vary across forest types, pixel independence is assumed for tractability; if spatial covariance is positive, the aggregated uncertainty could be larger, making the estimate conservative. The result from variance propagation can thus be interpreted as a conditional lower bound of regional uncertainty. To more robustly capture the instability of class−representative values under small-sample conditions, a bootstrap resampling pathway is implemented. First, the effective area of each class within the forested region is calculated by summing the posterior probabilities of all pixels for that class. As shown in Equation 10, the effective area of class k is estimated as the probability-weighted sum of pixel areas:

(10) $$S_{k}=\sum_{x}A_{x}p_{k}\left(x\right)$$

Subsequently, the class-specific representative carbon density is treated as a random variable supported by the sample plots. By performing bootstrap resampling on the within-class plot distributions or by directly perturbing the estimated $\mu_{k}$, a set of bootstrapped representative values $\mu_{k}^{\left(b\right)}$is generated. As shown in Equation 11, the total county-level carbon stock for the b-th bootstrap iteration is calculated by summing the products of class area and the corresponding bootstrapped representative value across all classes:

(11) $$T^{\left(b\right)}=\sum_{k}S_{k}\mu_{k}^{\left(b\right)}$$

Repeating this procedure over a large number of bootstrap iterations yields an empirical distribution of the total carbon stock T, from which confidence intervals are derived (David et al., 2024; Qiu et al., 2024). In contrast to the analytical variance propagation, the bootstrap approach places greater emphasis on holistically capturing parameter uncertainty-particularly the instability of class-representative values-under small-sample conditions. This combination of an analytical (variance propagation) and a resampling-based (bootstrap) strategy provides complementary perspectives. Consequently, the framework not only delivers pixel-level uncertainty maps but also offers a multi-faceted assessment of the robustness of the regional AGCS total estimate (Araza et al., 2022).

## 3 Results

### 3.1 Plot-level AGCS characteristics and three-class stratification

**Figure 3:**
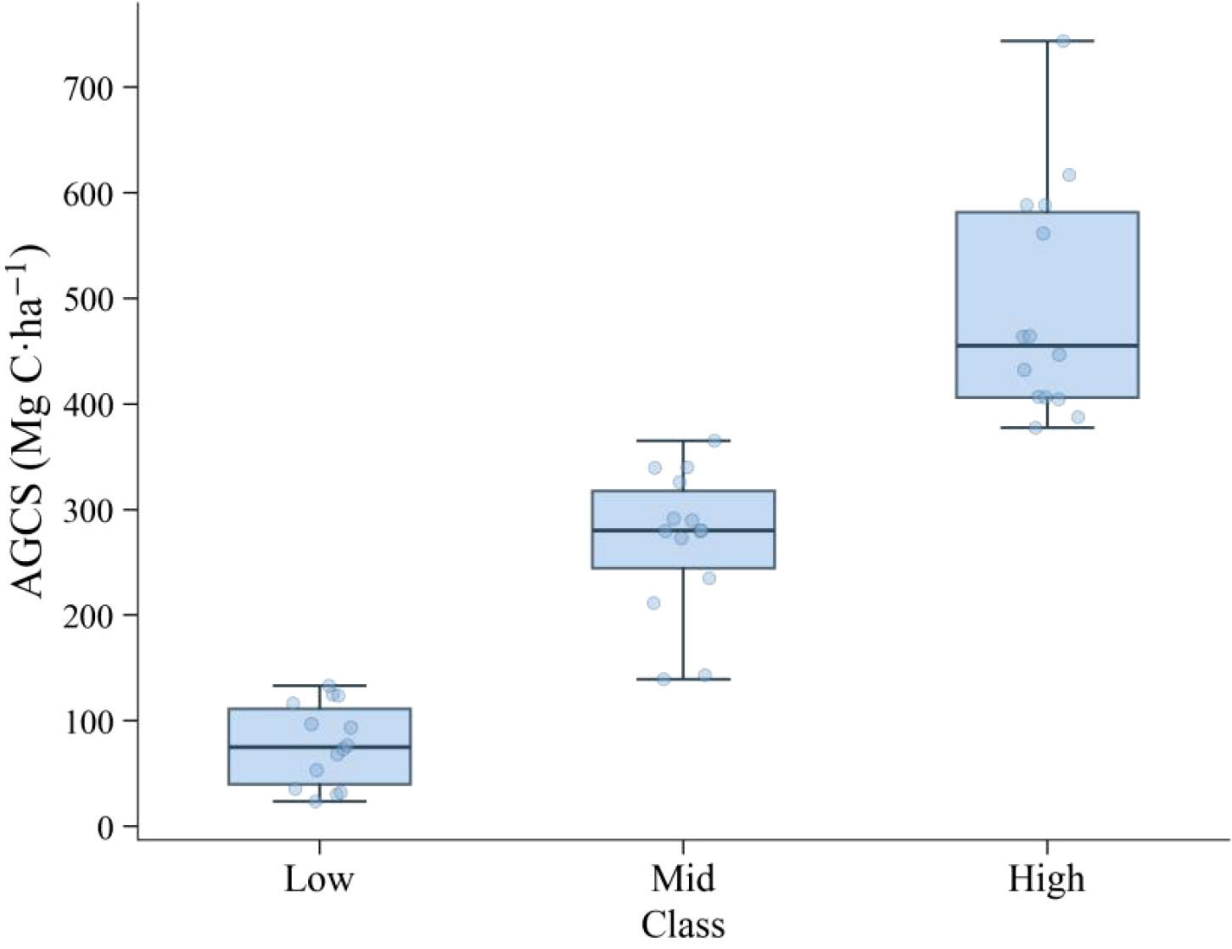
Boxplots of expected aboveground carbon stock (AGCS) grouped by predicted class (Low, Mid, High).

The AGCS of the 42 sample plots in Huize County, after scale harmonization and correction, ranged from 23.39-743.55Mg C·ha^-1^. Based on the 25th and 75th percentiles of the plot-level AGCS distribution, the plots were divided into three tiers: Low, Mid, and High, each containing 14 plots. The Low, Mid, and High tiers correspond to the 0-25th, 25-75th, and 75-100th percentile intervals, respectively. The median AGCS of each tier increased monotonically with tier rank (**Figure 3**). The representative carbon density for each tier was defined as the median AGCS of its constituent plots:  μ_Low_ = 75,  μ_Mid_= 280, μ_High_= 455 Mg C·ha^-1^. These values satisfy the order μ_Low_ <μ_Mid_ <μ_High_, thereby providing the ordered representative values required for the subsequent probability-expectation fusion.

### 3.2 Accuracy and discriminative performance of the probabilistic soft-classification model

#### 3.2.1 Classification performance under spatial cross-validation

Under the 2×2 spatial block cross-validation scheme, the LightGBM three-class model was evaluated using multi-source remote sensing and topographic predictors. Overall performance was modest (**Table 4**), with OA = 0.381, Macro-F1 = 0.377, Cohen’s Kappa = 0.071, and quadratic-weighted Kappa = 0.241. The AUC was 0.584 for the Low class and 0.518 for the High class, suggesting limited class-level separability under spatial extrapolation. Results were consistent under leakage-free within-fold feature screening (Corr-pruned (0.98) and PermTop-20), and the full feature set was retained for the main analyses.

**Table 4:** Classification performance under 2×2 spatial block cross-validation.

CV scheme	Feature setting	OA	Macro-F1	Kappa	Kappa (quadratic)	AUC (Low vs rest)	AUC (High vs rest)
2×2 Spatial block	All features	0.381	0.377	0.071	0.241	0.584	0.518
	Corr-pruned (0.98)	0.381	0.372	0.071	0.167	0.577	0.505
	PermTop-20	0.333	0.322	0.012	0.133	0.551	0.497

Corr-pruned (0.98) removes one feature from each highly correlated pair ( |ρ| > 0.98, Pearson). PermTop-20 retains the top-20 features ranked by permutation importance. All screening is performed within each training fold to avoid information leakage.

**Figure 4:**
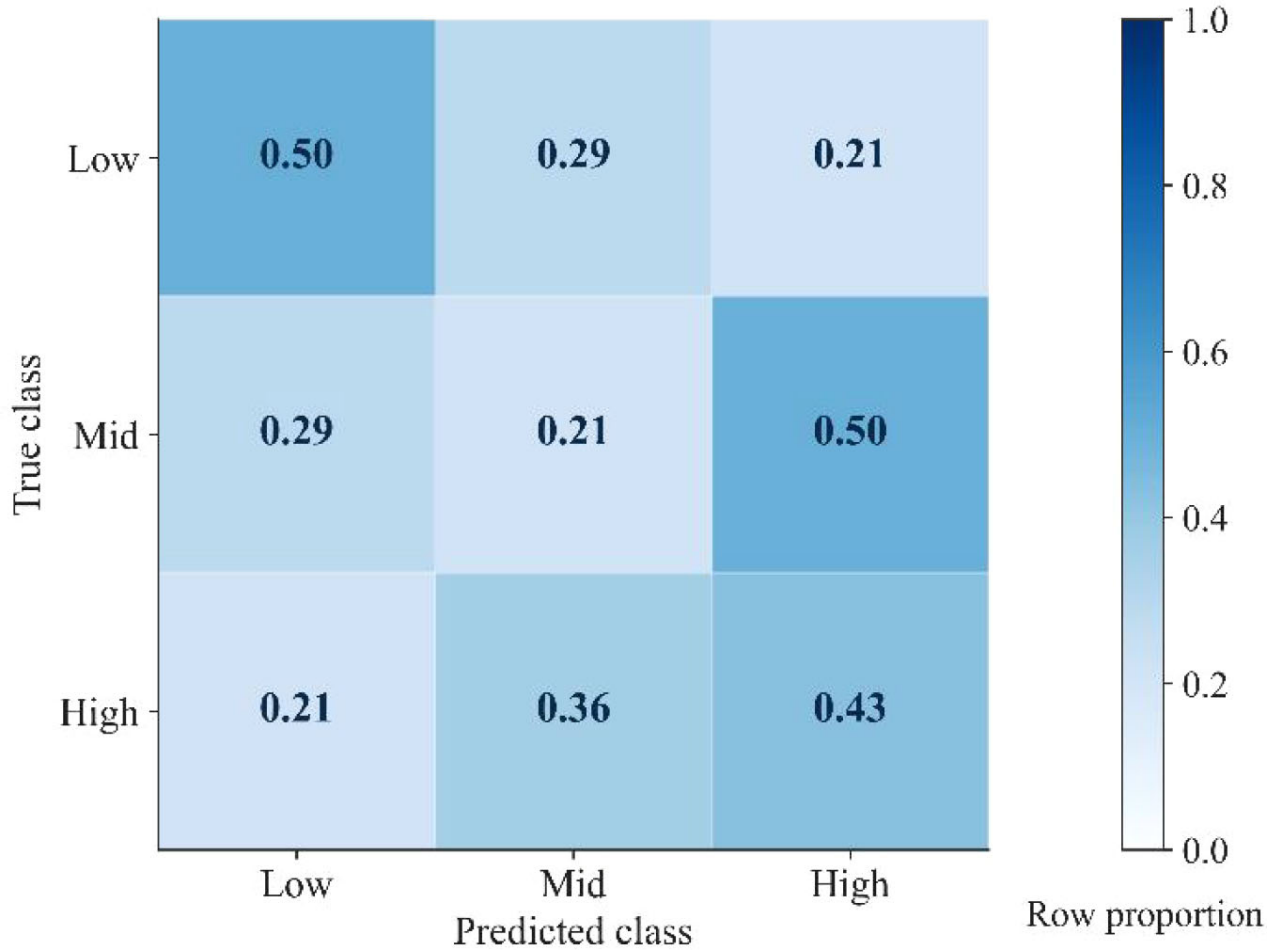
Row-normalized confusion matrix (row proportions) for the three-class model evaluated using 2×2 spatial block cross-validation.

The row-normalized confusion matrix (**Figure 4**) further shows that recall was approximately 0.50, 0.21, and 0.43 for the Low, Mid, and High classes, respectively. Misclassifications occurred mainly between adjacent tiers, with the Mid class most frequently confused with the High class.

#### 3.2.2 Consistency between predicted classes and the carbon density gradient

**Figure 5:**
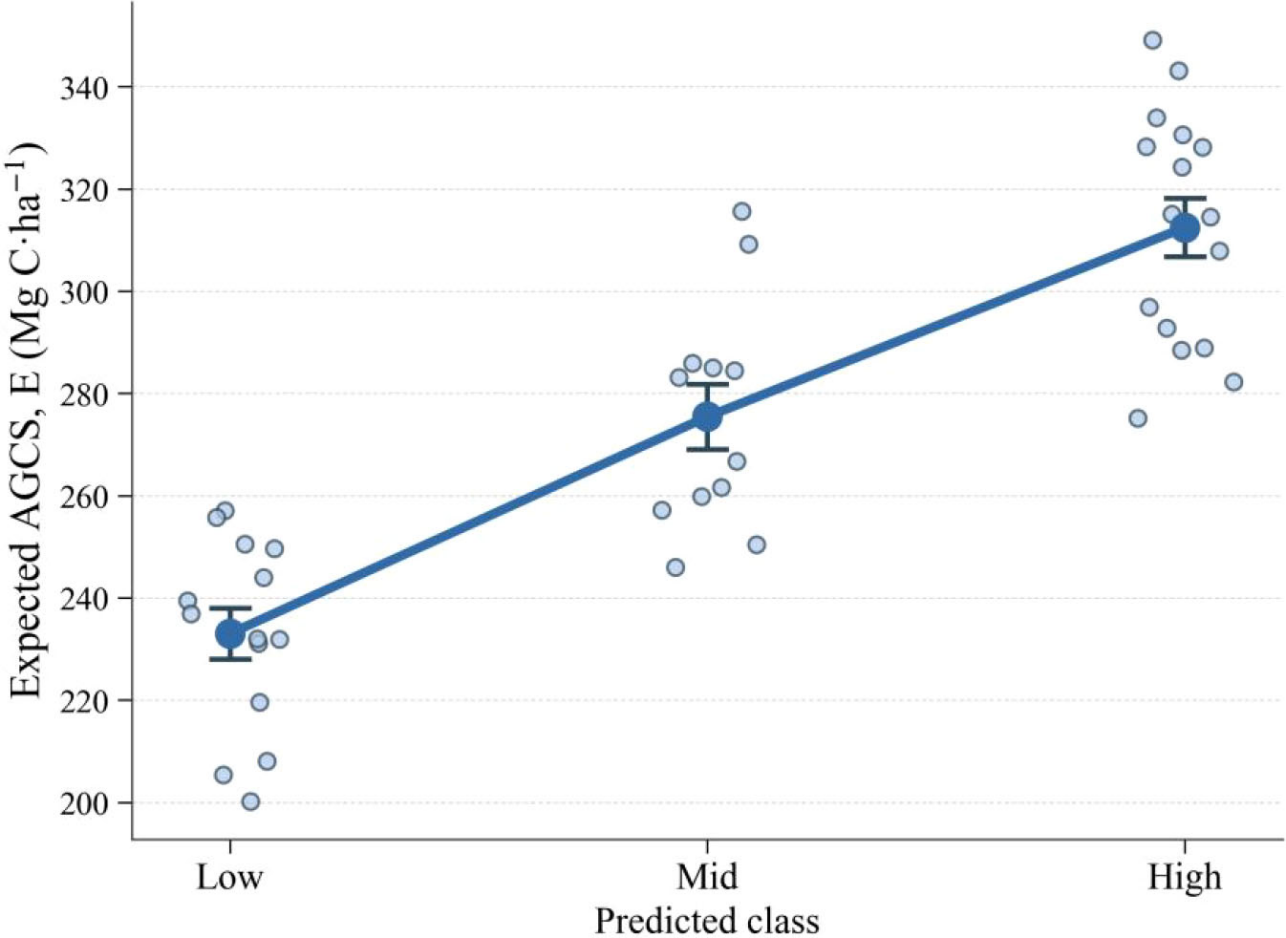
Mean out-of-fold expected AGCS by predicted class (Low, Mid, High), showing a monotonic increase in carbon density.

Based on the out-of-fold posterior probabilities, the expected AGCS was computed for each sample plot and then aggregated by its predicted class. The mean expected carbon densities for plots predicted as Low, Mid, and High were approximately 213.0, 288.3, and 332.4 Mg C·ha^-1^, respectively. These results demonstrate a monotonic increase in the estimated AGCS with ascending predicted class, which is consistent with the predefined ordinal structure of the classes (**Figure 5**).

#### 3.2.3 Out-of-fold baseline comparison for continuous AGCS estimation

Under the rigorous 2×2 spatial block cross-validation, probability expectation fusion yielded lower overall errors in continuous carbon-density estimation than both hard class assignment and a direct regression baseline (**Table 5**). Probability expectation fusion achieved an RMSE of 182.13 Mg C·ha^-1^, an MAE of 150.92 Mg C·ha^-1^, and a bias of -4.64 Mg C·ha^-1^, compared with 207.38/162.47/-1.32Mg C·ha^-1^ for hard class assignment and 196.77/154.32/15.20 Mg C·ha^-1^ for the direct regression baseline (LightGBM regressor) trained on the same predictors.

**Table 5:** Comparison of continuous estimation errors among probability expectation fusion, hard class assignment, and direct regression.

Method	RMSE	MAE	Bias
Soft expectation	182.13	150.92	-4.64
Hard assignment	207.38	162.47	-1.32
Direct regression	196.77	154.32	15.20

**Figure 6:**
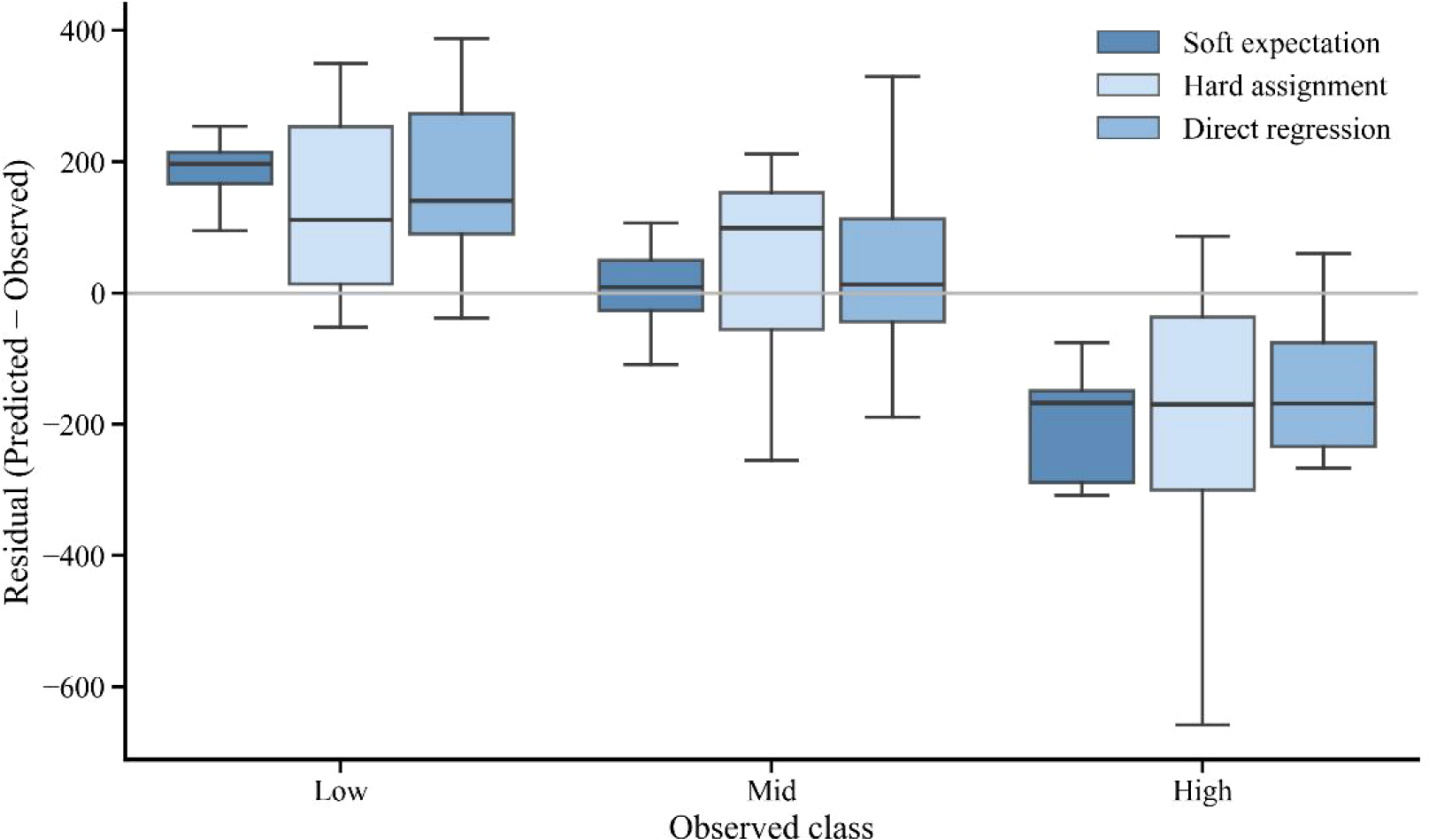
Out-of-fold residual distributions (predicted-observed AGCS) by observed class, comparing probability-expectation fusion (soft expectation), hard assignment, and direct regression.

The out-of-fold residual distributions (predicted-observed) further highlight differences in error structure across the Low, Mid, and High tiers (**Figure 6**), with the probabilistic method showing a generally narrower spread, particularly for the High tier.

### 3.3 Probability-weighted continuous AGCS estimates

**Figure 7:**
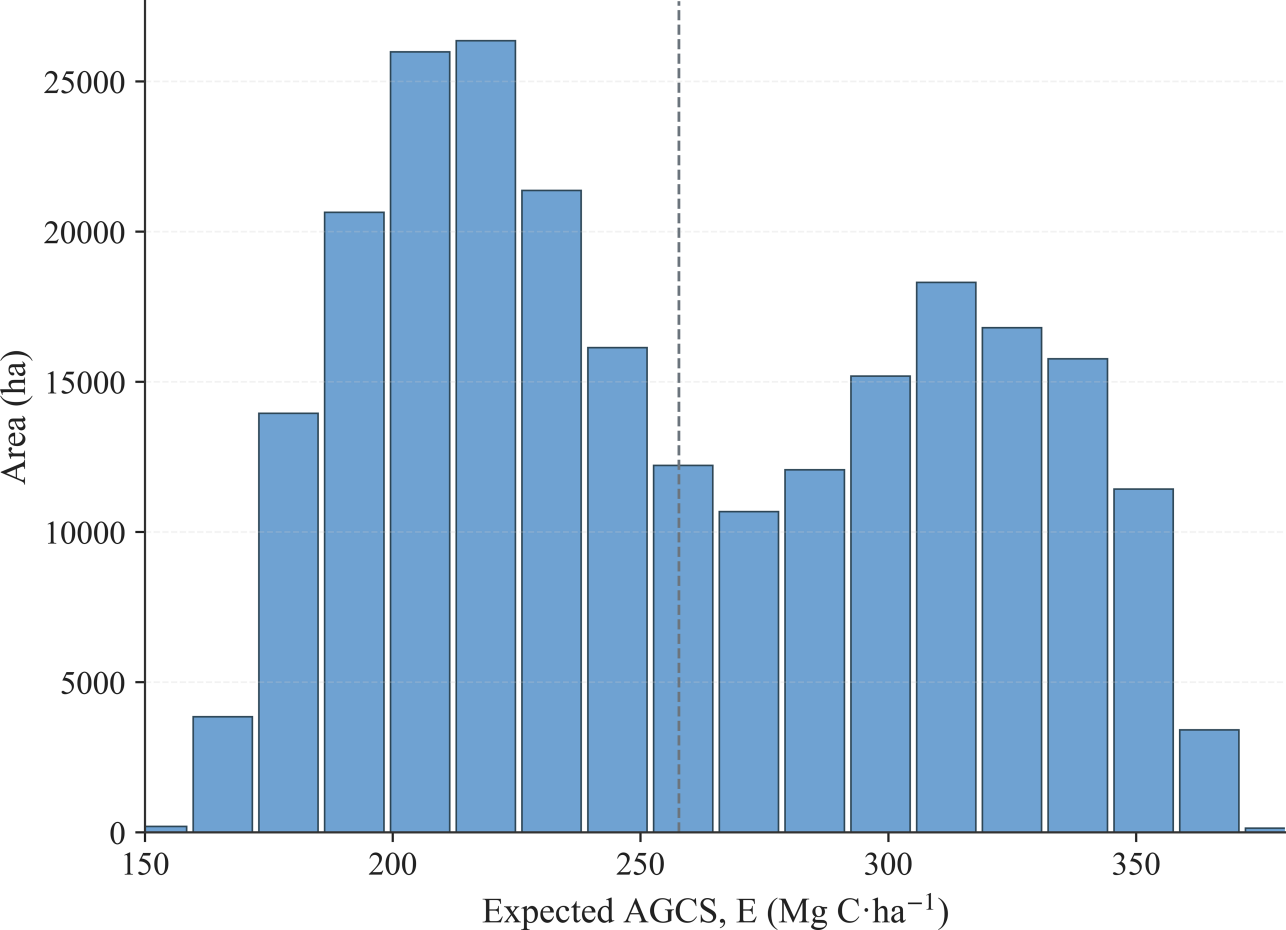
Histogram of expected AGCS across forest pixels (area-weighted).

**Figure 8:**
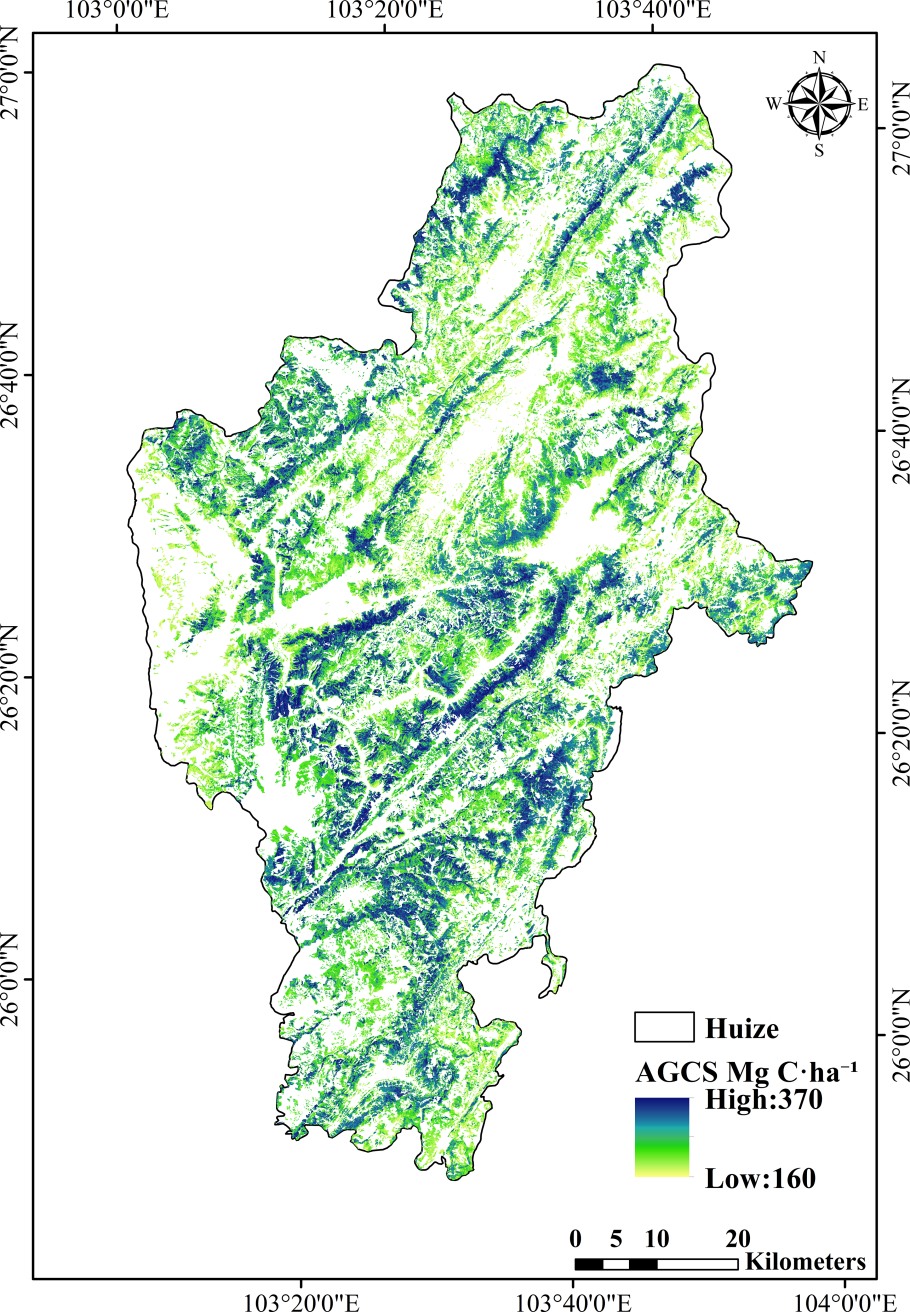
Spatial distribution of probability-weighted expected AGCS in Huize County.

After obtaining the posterior probability maps for the Low, Mid, and High tiers and their class-specific representative carbon densities, a continuous AGCS value was calculated for each forest pixel using probability−weighted expectation. Within the forest mask, approximately 2.04 × 10^6^ valid pixels (corresponding to an area of about 1.84 × 10^5^ ha) were processed. The area−weighted mean of the pixel−level AGCS was approximately 263.2 Mg C·ha^-1^. The histogram of the expected AGCS is shown in **Figure 7**, and its spatial distribution is presented in **Figure 8**. Spatially, higher AGCS values are primarily concentrated in the continuous forest areas from the central to northwestern parts of the county. In contrast, lower values are more frequently found in the southeastern region and in areas with fragmented forest patches or edges. Since the expected AGCS at each pixel is a convex combination of the three class-specific representative values *(*  μ_Low_, μ_Mid_, μ_High_), the theoretical range of pixel values is bounded by  μ_Low_, μ_Mid_, μ_High_.

### 3.4 Spatial distribution of prediction uncertainty

**Figure 9:**
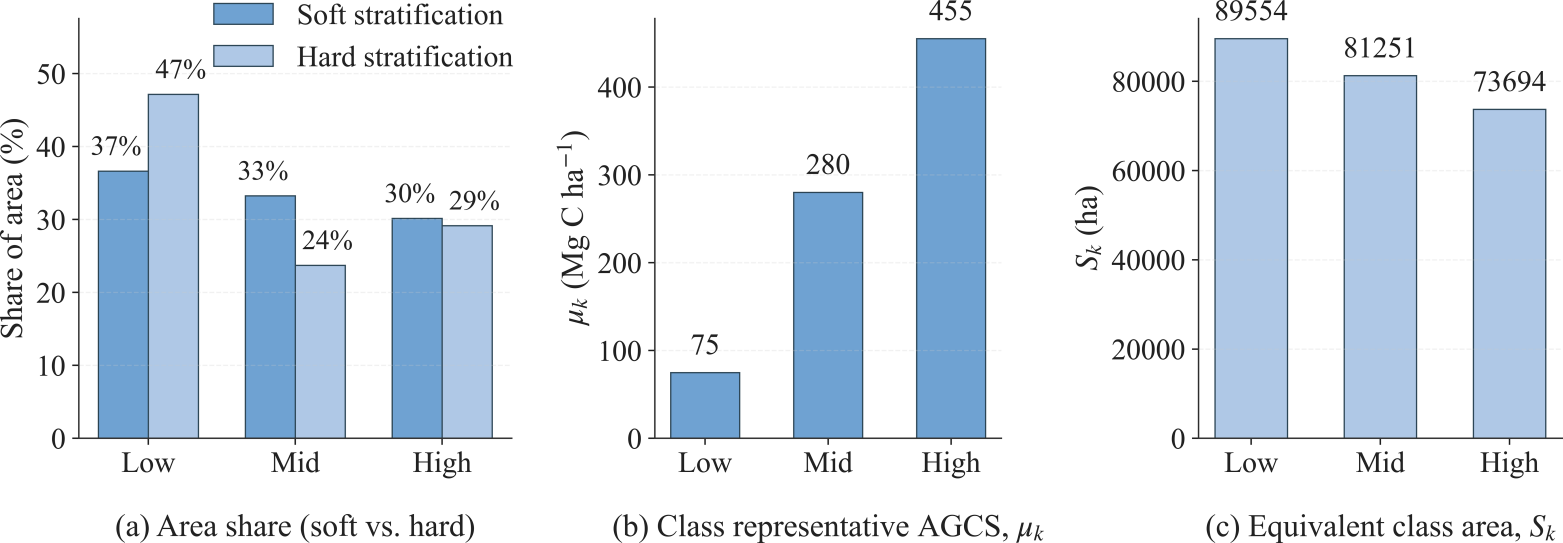
Stratification and decomposition of county-scale AGCS by carbon tier. **(a)** Comparison of class area shares under soft (probabilistic) and hard stratification within the forested area; **(b)** Class representative AGCS, ${\text{μ}}_{\text{k}}$ (Mg C·ha^-1^); **(c)** Effective class area, ${\text{S}}_{\text{k}}$ (ha), for the three tiers (Low, Mid, High).

Within the forested area, the proportional area of the Low, Mid, and High tiers was quantified using two approaches: (1) soft classification, based on the probability-weighted effective area, and (2) hard classification, based on the argmax class assignment (see **Figure 9a**). The results indicate that soft classification yields relatively balanced area shares across the three tiers, whereas hard classification reduces the Mid-tier share and increases the Low-tier share. Furthermore, the contribution of each tier to the total regional carbon stock was calculated as $T_{k}=S_{k}\mu_{k}$, where $S_{k}$ is the effective area of tier k (**Figure 9c**) and $\mu_{k}$ is its representative carbon density (**Figure 9b**). The total stock T is the sum of the tier-level contributions: $T=\sum_{k}T_{k}$. The decomposition reveals that the High tier contributes the most to the total stock, followed by the Mid tier, with the Low tier contributing the least.

### 3.5 County-scale forest AGCS totals and uncertainty estimates

The total forest AGCS in Huize County was estimated by spatially integrating the expected AGCS raster over the forested area, yielding a value of approximately 4.85 × 10^7^ Mg C. This total is consistent with the sum calculated from tier-specific effective areas and representative values. with only minor discrepancies attributable to numerical rounding. Uncertainty in this regional total was quantified using two complementary approaches. The variance propagation method produced a standard error of about 1.99 × 10^4^ Mg C, corresponding to a 95% confidence interval (CI) of 4.84 × 10^7^–4.85 × 10^7^ Mg C. In contrast, the bootstrap resampling method yielded a mean total estimate of 4.86 × 10^7^ Mg C, with a 95% CI of 4.54 × 10^7^–5.18 × 10^7^ Mg C.

For context, an independent estimate of the county’s aboveground forest carbon stock was derived from the Second Forest Resource Inventory data, applying the same biomass-to-carbon conversion factor. This external estimate is approximately 4.43 × 10^7^ Mg C, which is of the same order of magnitude as the results from this study, providing a separate validation of the proposed framework’s plausibility.

## 4 Discussion

### 4.1 Rationale and advantages of the probabilistic soft-classification–expectation fusion framework

In scenarios with sparse samples and highly heterogeneous terrain, direct continuous regression on AGCS often yields unstable fits and may produce extrapolative predictions outside the training domain. This study reframes the problem as hierarchical classification with expectation fusion: first, multi-source remote-sensing and topographic features are used to classify three tiers (Low, Mid, and High); then continuous AGCS is generated by probability-weighting tier-specific representative values using posterior probabilities. This shifts the modeling focus from estimating precise point values to recovering ordinal gradients, better aligning with the information obtainable under small-sample conditions.

This framework shows internally consistent behavior across three dimensions: ranking, spatial patterns, and regional aggregate totals. Predicted grades correspond to measured AGCS values in a monotonic increasing sequence. Continuous AGCS maps exhibit gradual gradients along topography and forest distribution, avoiding large-scale artifactual discontinuities. At the county scale, grid-based integration is consistent with totals derived from representative values × area-equivalent extent, supporting the pixel-to-region aggregation pipeline. Under the same 2×2 spatial block cross-validation, probabilistic expectation fusion consistently achieves lower continuous-estimation errors and a smaller systematic bias than both the hard-label classification baseline and direct continuous regression, supporting expectation fusion as a practical strategy for generating continuous AGCS under strict spatial extrapolation.

### 4.2 Ecological interpretation of multi-sensor remote sensing features and topographic gradients

Optical, radar, and topographic features correspond to three ecological dimensions in this study: canopy state, stand structure, and site conditions. Landsat spectral data and vegetation indices characterize leaf area, greenness, and canopy cover; ALOS PALSAR backscatter and texture reflect canopy roughness and woody biomass; elevation, slope, and aspect derived from DEMs represent hydrothermal conditions, erosion risk, and solar-radiation regimes. Together, these feature groups constrain the potential upper bounds of forest growth and current structure, enabling the model to recover ecologically plausible carbon-density gradients even with limited plot data.

Regarding management implications, the grade-probability field, combined with a decomposition of intensity ( $\mu_{k}$) × equivalent area ( $S_{k}$), clarifies how different grades contribute to county-level totals. High-carbon grades often correspond to mature forests with favorable site conditions and complex structures and therefore represent priority areas for protection and degradation avoidance. Medium-carbon grades typically occupy larger equivalent areas, indicating the primary potential to increase carbon storage per unit area through tending and structural optimization. Low-carbon grades predominantly indicate sparse, degraded, or site-constrained stands, providing spatial cues for restoration and conversion. Consequently, the outputs support carbon accounting and applications such as carbon trading and ecological restoration. The uncertainty-bounded totals enable MRV/baseline reporting, while the grade-probability field supports restoration prioritization and protection zoning.

### 4.3 Implications of the spatial cross-validation strategy and its results

This study employs 2×2 spatial block cross-validation to evaluate the performance of hierarchical classification models under spatial extrapolation scenarios, rather than optimizing classification metrics on highly overlapping samples. This configuration forces the models to confront previously unseen spatial regions, thereby yielding performance estimates that are closer to real-world mapping conditions (Roberts et al., 2017; Ploton et al., 2020).

Under this stricter evaluation framework, OA, Macro-F1, and Kappa remain modest, reflecting the inherent difficulty of distinguishing three carbon tiers in a small-sample, highly heterogeneous mountainous area. Out-of-fold results nonetheless show a consistent error structure in the confusion matrix: the Mid tier is most difficult to separate and tends to be confused with adjacent tiers, which is consistent with its role as a transitional zone along the carbon gradient. Accordingly, these conservative metrics can be interpreted as a lower-bound indication of extrapolation performance under spatially disjoint training–application conditions, while the resulting maps remain informative for describing broad carbon-gradient patterns and constraining county-scale totals. The one-vs-rest AUC values for the extreme classes (Low: 0.584; High: 0.518) are close to 0.5, indicating limited class-level discrimination under strict spatial extrapolation. This is consistent with the confusion matrix, where overlap in feature space—especially for the transitional Mid tier—limits separability and motivates using probabilistic expectation fusion for continuous reconstruction.

### 4.4 Limitations and potential improvements

It is important to emphasize that the differing widths of the uncertainty paths do not contradict each other; rather, they reflect different sources and emphases of uncertainty. Pixel-level variance propagation primarily captures the combined effects of class probabilities and within-class variability. When aggregated to the regional scale, it adopts the simplifying assumption that cross-pixel covariance is negligible. Consequently, its county-level intervals serve as a reference lower bound for overall uncertainty, making this approach well suited to identifying high-uncertainty patches and optimizing monitoring-site selection. Regional bootstrap explicitly incorporates the sampling variability of the three-tier representative values under small sample sizes, yielding more conservative intervals. For comparisons of county-level total carbon and tier contributions in management reporting and decision-making, this study recommends using the bootstrap-based 95% confidence interval as the primary reporting interval (Clough et al., 2016).

Although this framework enables spatial mapping and uncertainty quantification under limited plot conditions, several limitations warrant clarification. In addition to sampling limitations, uncertainties in the input predictors can affect local AGCS estimates. Landsat 5 observations may retain residual atmospheric noise and cloud/shadow artifacts after masking, and the 30-m resolution can introduce mixed-pixel effects in topographically complex landscapes; temporal mismatch between plot measurements and image acquisition can further weaken predictor–response consistency. For ALOS PALSAR, speckle noise and potential signal saturation in dense canopies may reduce sensitivity at higher carbon densities, contributing to overlap among tiers. Model uncertainty may also be influenced by hyperparameter choices and the stability of feature importance under small samples, although all comparisons in this study were conducted under the same leakage-free fold-wise setting. First, the number and representativeness of plots remain limited, and plot acquisition years may not fully match the image acquisition years. Future work could optimize plot placement using joint stratification by forest type and topographic gradient, prioritizing additional samples from high-carbon, low-carbon, and transitional zones to improve extrapolation robustness. Second, although three-tier quantile segmentation promotes class balance, the mid tier inevitably overlaps with the extremes in feature space, resulting in low recall for the mid tier. To support finer management zoning, potential extensions include unequal-width quantiles, local grade refinement, and segmentation rules that incorporate forest type and site conditions, thereby improving separability of the target classes. Third, the current model-data configuration is limited to a single period with multi-source predictors; it does not explicitly leverage time-series information or systematically compare the proposed ordinal classification with spatial statistical or Bayesian hierarchical models. Future work should integrate class probabilities, sample representativeness, and spatial correlation structures within a unified modeling framework, and extend the approach to dynamic monitoring using multi-temporal remote sensing.

Additionally, the county-level totals estimated in this study are slightly higher (approximately 10%) than those reported by the second-class survey. This discrepancy may stem from differences in forest-area delineation, forest-type classification, carbon-factor assumptions, and temporal scope, highlighting the need for joint assessment and recalibration using updated inventory data and more detailed stand-level information.

### 4.5 Comparison with previous studies and methodological contributions

Most regional AGCS studies map continuous stocks via direct regression using multi-source remote-sensing predictors, sometimes combined with forest-type stratification, and report performance mainly from random cross-validation or overall error statistics (Lu et al., 2025; Wu et al., 2025). In small-sample mountainous settings, such designs can be sensitive to spatial leakage and provide limited guidance on where predictions are unreliable (Roberts et al., 2017). In contrast, this study models AGCS as three ordinal tiers and evaluates the model using spatial block cross-validation; continuous AGCS is then reconstructed through probability–expectation fusion rather than hard labels. The framework further reports uncertainty from pixel-level variance propagation to county-scale bootstrap intervals, enabling uncertainty-aware carbon accounting and supporting applications such as carbon trading and ecological restoration in data-scarce regions. Methodologically, the framework addresses limited plot density by using hierarchical classification to characterize carbon gradients and by applying probability–expectation fusion to reconstruct continuous fields and regional totals, balancing statistical stability with spatially continuous representation.

Compared with conventional approaches, this study makes three contributions: (1) it proposes a probabilistic soft classification–expectation-fusion pathway to mitigate hard boundary artifacts in transition zones; (2) it develops a dual-path uncertainty assessment that combines variance propagation and bootstrap methods to provide closed-loop quantification and interval reporting from pixel level to county totals; (3) it employs spatial block cross-validation to characterize extrapolation performance and reduce spatial leakage caused by random partitioning, thereby producing evaluations that are more representative of real-world applications.

## 5 Conclusion

This study aimed to develop a spatially explicit and uncertainty-aware framework for continuous estimation and mapping of forest aboveground carbon storage (AGCS) in data-scarce, topographically complex mountainous regions using multi-source remote sensing and limited field plots.

(1) For mountainous counties with sparse samples and highly heterogeneous terrain, this paper proposes a probabilistic soft-thresholding–expectation-fusion framework tailored to small-sample estimation of forest aboveground carbon storage (AGCS). Using Landsat 5 optical imagery, ALOS PALSAR radar data, and DEM-derived topographic variables as predictors, we construct a probabilistic soft-classification model for Low/Mid/High AGCS classes. By integrating class representative values and monotonicity constraints across grades, the probability field is transformed into a continuous AGCS estimate, achieving a balance between statistical robustness and ecological interpretability.

(2) Using 2×2 spatial block cross-validation, we systematically evaluated the performance of the three-class model under spatial extrapolation. Although overall accuracy remained moderate under strict spatial constraints, the median AGCS values of plots assigned to each predicted grade increased monotonically across grades, suggesting that the model can recover ecologically meaningful carbon-density gradients from multi-source remote-sensing and topographic features. At the continuous-estimation stage under the same spatial CV, probability expectation fusion yielded lower prediction errors and a smaller systematic bias than both the hard-label assignment baseline and a direct regression baseline, supporting expectation fusion as a conservative strategy for generating continuous AGCS when plot support is limited. (3) At the county scale, both approaches-the probability-weighted continuous AGCS grid and the” ${\text{μ}}_{\text{k}}$- ${\text{S}}_{\text{k}}$“decomposition-yielded similar estimates of total forest AGCS carbon stocks for Huize County (≈ 4.85 × 10^7^ Mg C). The corresponding 95% confidence intervals were 4.84 × 10^7^–4.85 × 10^7^ Mg C and 4.54 × 10^7^–5.18 × 10^7^ Mg C, respectively. The close agreement of the point estimates and the overlap of the confidence intervals indicate a numerically closed-loop estimation process from pixel to county scale, demonstrating robust total estimates under different uncertainty assumptions.

Overall, the probabilistic soft-classification–expectation-fusion plus dual-path uncertainty-assessment framework developed in this study enables spatially continuous mapping of forest AGCS, characterization of grade distributions, and estimation of county-level totals with associated confidence intervals for Huize County, even with limited plot availability. This framework provides a structurally transparent and uncertainty-aware technical pathway for county-scale ecological monitoring and forest carbon management. This method relies on multi-source remote-sensing and topographic features and uses a relatively simple model structure with clear physical interpretability. It is potentially applicable to mountainous forest areas where plot data are scarce but remote-sensing data are relatively abundant. Future work could improve the accuracy and spatiotemporal adaptability of AGCS estimation by expanding the plot network and incorporating more refined stratification schemes and spatial statistical models.

## Data Availability

The raw data supporting the conclusions of this article will be made available by the authors, without undue reservation.
